# A survey of referee participation, training and injury in elite gaelic games referees

**DOI:** 10.1186/1471-2474-10-74

**Published:** 2009-06-22

**Authors:** Catherine Blake, James Sherry, Conor Gissane

**Affiliations:** 1 UCD School of Physiotherapy and Performance Science, University College Dublin, Belfield, Dublin 4. Ireland; 2 Department of Human Sciences, St. Mary's University College, Strawberry Hill, Twickenham, Middlesex, UK

## Abstract

**Background:**

Referees in Gaelic games are exposed to injury risk in match-play and training. Little is currently know about the degree of exposure or the prevalence of injury in this group. The aim of this study was to determine the time commitment to refereeing and training in elite-level Gaelic referees and to establish, for the first time, point and period (past 12 months) prevalence of Gaelic games injury in these officials.

**Methods:**

A retrospective survey was posted to the complete list of 111 male referees who officiated in elite-level competition in Gaelic football and hurling at the end of the 2005 competition season. Data were summarised using percentages with 95% Confidence Intervals.

**Results:**

The response rate was 80% (n = 89). Mean age was 42 ± 6 years, ranging from 28–55 years. Forty eight percent were football referees, 25% were hurling referees and 27% refereed both football and hurling. Most referees (69%) officiated at 3–4 games weekly (range 1–6) and most (62%) trained 2–3 times per week (range 1–7). Fourteen percent (n = 12) were currently injured (95% CI 9–21%). Annual injury prevalence was 58% (95% CI 46 to 70%) for football, 50% (95% CI 33 to 67%) for hurling and 42% (95% CI 27 to 58%) for dual referee groups. Sixty percent of injuries were sustained while refereeing match play. The majority (83%, n = 40) were to the lower limb and the predominant (56%, n = 27) injury mechanism was running or sprinting. The most prevalent injuries were hamstring strain (n = 12, 25% of injuries) and calf strain (n = 9, 19% of injuries). Injury causing time off from refereeing was reported by 31% of all referees (95% CI 24 to 40%, n = 28), for a median duration of 3 weeks.

**Conclusion:**

Participation in official duties and training is high in elite Gaelic games referees, despite the amateur status of the sports. Gaelic games injury is common in the referee cohort, with lower limb injury predominating. These injuries have implications for both the referee and for organisation of the games.

## Background

Referees and umpires are a part of all sporting events. In field games, they are required to keep up with play and adjudicate decisions involving breaches of the rules. Some attention has been given to the physiological demands placed on soccer [1-3] and rugby referees [4] in particular, but there are few published studies on referee injury. The most recent relate to officials selected for the 2006 FIFA Soccer World Cup [5] and the 2007 FIFA Women's World Cup [6]. Earlier reports have concerned injury in Australian Football League (AFL) umpires [7] and overuse injury in soccer referees during a single tournament [8].

Gaelic football and hurling are the two national games of Ireland, governed by a central body, the Gaelic Athletic Association (GAA) which has international branches in the UK, Europe, North America and Australasia. Both sports involve games of up to 70 minutes, played by 2 opposing teams of 15 players. Games are adjudicated by an on-field match referee, supported by linesmen and goal-end umpires. Gaelic football is played with a round ball, with similarities to AFL, while hurling is played with a stick and ball, similar to field hockey or shinty. Both Gaelic football and hurling are high velocity, multidirectional contact sports, which demand sprinting, endurance and quick directional change from both the players and the match referee. Participation ranges from club level competition based primarily within county divisions, to elite inter-county league and championship competitions, Despite near professional training and match schedules at the highest level, these sports continue to retain amateur status.

In Gaelic games, similar to other sports, the officials are overlooked when research is carried out. Very little has been published with regard to their time commitment due to their official duties and training. These individuals are amateurs who voluntarily give their time and effort to the sport, as are the players, but they are required to carry out their official duties at all levels of the game, and without at least one present, a game cannot take place. To carry out their duties effectively requires a level of fitness and cognitive demand, while at the same time exposing them to certain injury risk. Both of these elements may vary depending upon the standard of the game of which they have been put in charge. The elite referees studied here are selected annually to form a panel to adjudicate at the highest level competition, based upon consistency of performance during regular assessment. These individuals are allocated inter-county fixtures, while at the same time continuing to officiate at club-level competitions in their base county.

Whilst the injury risk to players in Gaelic games has been examined [9], the prevalence and risk of injury to which Gaelic games referees are exposed has not. It is an important aspect to quantify as it can have implications, both in terms of time lost from refereeing, and in terms of affecting their ability to carry out their normal activities and employment.

The purpose of this research was to describe the time commitment to refereeing and training of elite referees in Gaelic Football and Hurling (frequency of refereeing, type and frequency of training). It further sought to record the frequency, type and consequences of injury (time lost from refereeing, time to recover, impact on work and daily activities, treatment required) during the past year. The focus was Gaelic games related injury sustained in participation or training for official duties.

## Methods

A census of inter-county referees in Gaelic hurling and football was undertaken towards the end of the inter-county championship season in September 2005 using a postal survey. This asked respondents to recall refereeing participation and injuries sustained during the prior 12 months. This retrospective self-report methodology has limitations [10], thus it was considered that the appropriate injury measurement variables were period (annual) and point (current) prevalence. Ethical approval was granted by the university ethics committee.

### Subject Recruitment

A complete list of all one hundred and eleven referees at inter-county grade was obtained from the National Referees co-ordinator in central GAA administration. All of the referees were male. Each individual on the list was posted a questionnaire pack, containing an explanatory letter requesting them to return the questionnaire anonymously in a pre-paid envelope. Reminder letters were sent after two weeks.

### Injury definition

An injury was described as 'any injury that caused pain or interference with normal activities for a period of 24 hours or more'. This threshold for reporting an injury was chosen in the absence of a consensus injury definition for these sports at the time of the study. The minimum 24 hour time frame was used to ensure that only injuries causing impairment of at least this duration were included. The use of 'games lost' from refereeing as a criterion in the definition was not feasible, since there can be a large variance in the interval between games for referees, dependant on allocation of game fixtures.

### Questionnaire

The questionnaire was designed to seek information on; (i) demographic details, (ii) level of participation in refereeing, (iii) injury episodes, mechanism and consequences and, (iv) training regimes. It was piloted on a group of 28 Gaelic football club-level referees in County Fermanagh prior to the main study, to ensure acceptability and clarity. Feedback on face and content validity was sought from the pilot study and from a panel consisting of a sports physician, physiotherapists and a referee. Closed and open-ended questions were used to gather quantitative and qualitative data. No inter-county referees were included in the pilot study.

### Statistical analysis

Data were coded and entered onto Microsoft Excel, SPSS version 12 and VRP Injury Statistics Software for analysis [11]. Results are presented as percentages with 95% confidence intervals, expressed as either a percentage of all respondents, as a percentage of those injured in the past year, or as a percentage of injuries, as appropriate. Responses from open ended questions and other voluntary comments from respondents were transcribed and underwent content analysis for common themes.

## Results

### Demographics and level of refereeing activity

Eighty-nine inter-county referees out of 111 surveyed responded to the questionnaire, representing an 80% response rate, which was distributed through 29 of the 32 counties of Ireland. Table 1 illustrates age range and level of refereeing activity in the cohort. The mean age was 42 ± 6 (mean ± sd) years, ranging from 28 to 55 years. Forty three (48%) refereed football, 22 (25%) were hurling referees and 24 (27%) refereed both football and hurling. All referees participated in club level games in addition to inter-county duties and most referees (69%, 95% CI 60 to 76%) reported that they officiated 3–4 times each week, with a further 9% officiating 5 or more times weekly. Given the seasonal variation in refereeing frequency, a second question sought the number of games refereed in the preceding 4 weeks, where it was seen that 18% had officiated at 4 or more games per week. It was notable that 86% (95% CI 70 to 94%) and 88% (95% CI 73 to 95%) of those in the hurling and dual categories respectively, refereed more than twice per week, by comparison with 67% (95% CI 55 to 78%) of the football referees.

**Table 1:** Demographic characteristics and refereeing activity in Gaelic games referees

	**Total** **n = 89**		**Football** **n = 43**		**Hurling** **n = 22**		**Dual Football and Hurling** **n = 24**	
	n (%)	95% CI	n (%)	95% CI	n (%)	95% CI	n (%)	95% CI
Age range								
≤ 30 years	9 (10)	6 to 17	3 (7)	3 to 16	2 (9)	3 to 24	3 (13)	5 to 28
31–35 years	8 (9)	5 to 15	3 (7)	3 to 16	2 (9)	3 to 24	4 (17)	8 to 32
36–40 years	16 (18)	12 to 26	10 (23)	14 to 35	3 (14)	6 to 30	3 (13)	5 to 28
41–45 years	29 (33)	25 to 41	17 (40)	28 to 52	4 (18)	8 to 35	8 (33)	20 to 50
46–50 years	24 (27)	20 to 35	9 (21)	13 to 33	11 (50)	33 to 67	4 (17)	8 to 32
> 50 years	3 (3)	1 to 8	1 (2)	0.3 to 5	-	-	2 (8)	3 to 22
								
Refereeing participation*								
Club games	89 (100)	97 to 100	43 (100)	94 to 100	22 (100)	89 to 100	24 (100)	90 to 100
College games	64 (72)	64 to 79	32 (74)	62 to 84	15 (68)	51 to 82	17 (71)	54 to 83
Schools games	59 (66)	58 to 74	26 (60)	48 to 72	14 (64)	46 to 78	19 (79)	63 to 90
Ladies games	26 (29)	22 to 38	13 (30)	20 to 43	5 (23)	12 to 40	6 (25)	14 to 42
Other	6 (7)	4 to 13	1 (2)	0.3 to 5	2 (9)	3 to 24	5 (21)	11 to 37
								
**Frequency of refereeing**								
1–2 times/week	20 (23)	16 to 31	14 (33)	22 to 45	3 (14)	6 to 30	3 (13)	5 to 28
3–4 times/week	61 (69)	60 to 76	26 (60)	48 to 72	18 (82)	65 to 92	16 (67)	50 to 80
5–6 times/week	8 (9)	5 to 15	3 (7)	3 to 16	1 (4)	10 to 18	5 (21)	11 to 37
								
**Refereeing in past 4 weeks**								
0 games	4 (5)	2 to 10	1 (2)	0.3 to 5	3 (14)	6 to 30	-	-
1–4 games	25 (28)	21 to 37	14 (33)	22 to 45	5 (23)	12 to 40	5 (21)	11 to 37
5–8 games	29 (33)	25 to 41	13 (30)	20 to 43	9 (41)	26 to 58	8 (33)	20 to 50
9–12 games	14 (16)	10 to 23	7 (16)	9 to 28	3 (14)	6 to 30	4 (17)	8 to 32
13–16 games	11 (12)	8 to 19	5 (12)	6 to 22	2 (9)	3 to 24	4 (17)	8 to 32
17–20 games	3 (3)	1 to 8	2 (5)	2 to 13	-	-	1 (4)	1 to 17
21–24 games	3 (3)	1 to 8	1 (2)	0.3 to 5	-	-	2 (8)	3 to 22

*Varying degree of referee participation, percentages do not total 100%

All percentages rounded to whole number.

### Training type and frequency

When questioned about their training habits, 30% (95% CI 23 to 39%, n = 27) of all referees reported that they followed a specific prescribed fitness training programme at the time and 17% (95% CI 11 to 24%, n = 15) reported that they had a structured programme which was self-devised. The remainder had a more eclectic approach to training. Nine percent (95% CI 5 to 15%, n = 8) stated that they got their programme from a health centre/gym, 1% (95% CI, 0.3 to 5%, n = 1) from his local GAA club and 3% (95% CI, 1 to 8%, n = 3) reported that they got theirs from sources such as peers, physiotherapists and official GAA resources. Three percent (95% CI 1 to 8%) (n = 3) stated that they train once per week or less, 62% (95% CI 53 to 70%) (n = 55) said they train 2–3 times, 28% (95% CI 21 to 37%) (n = 25) reported 4–5 times and 7% (95% CI 4 to 13%) (n = 6) stated that they train 6–7 times per week during the GAA season.

As part of their aerobic training sessions, 98% (95% CI 93 to 99%) (n = 87) of all referees reported that they run, while 29% (95% CI 22 to 38%) (n = 26) cycle, 15% (95% CI 10 to 22%) (n = 13) swim and 10% (95% CI 6 to 17%) (n = 9) use rowing as a training method. Other methods of aerobic training included skipping, and using a cross trainer. Eighty four percent (95% CI 77 to 90%) (n = 75) of referees indicated that they undertake speed and sprint training and 23% (95% CI 16 to 31%) (n = 20) incorporate a strength programme with weights into their training regimes.

### Prevalence of Injury

#### Current Gaelic games-related injury

Twelve of the 89 referees reported that they were currently injured and all of these injuries were associated with Gaelic games refereeing or training related to refereeing, giving an overall point prevalence of 14% (95% CI 9 to 21%). The injuries sustained included knee ligament sprain (n = 4), achilles strain (n = 2), calf strain (n = 1), ankle sprain (n = 1), shin splints (n = 1), groin strain (n = 1) and rib fracture (n = 1). The injuries were incurred by 4 football referees (9% point prevalence, 95% CI 4 to 19%), 7 hurling referees (32% point prevalence, 95% CI 18 to 49%) and 1 individual who was a dual referee (4% point prevalence, 95% CI 1 to 17%). Five referees (6% of all referees, 95% CI 3 to 11%) were currently unable to participate in refereeing due to injury.

#### Annual injury prevalence and mechanism

Fifty-four referees indicated that they had been injured in the last 12 months, representing 61% (95% CI 52 to 69%) of the total respondents. Eight percent of referees (n = 4) incurred injuries at work, home or in other activities, with the remaining 50 referees having sport-related injuries. Of these, four sustained injury while participating themselves in recreational or competitive sport, leaving 46 referees with Gaelic games injury associated with official duties. Further injury analysis was confined to these 46 referees who suffered injury related to official duties, referred to here as 'Gaelic games injury'. Twenty five were football referees, 11 were hurling referees and 10 were dual referees, giving annual prevalence of 52% (95% CI 43 to 60%) for the total group, 58% (95% CI 46 to 70%) for football, 50% (95% CI 33 to 67%) for hurling and 42% (95% CI 27 to 58%) for dual referee groups. Table 2 illustrates prevalence data and injury details. Two of the 46 (4%, 95% CI 1 to 12%) referees reported receiving a Gaelic games injury to more that one body area, thus 48 injuries were sustained. The majority of injuries (60%, n = 29) related to refereeing match play, while 33% (n = 16) of injuries occurred in training. A further 2 injuries occurred while participating in linesman duties and 1 more during fitness assessment for refereeing. Four (8%) of the Gaelic games injuries related to external force or physical contact while refereeing. Two were sustained through collision with a player, another though being struck by a football, and one related to rib fracture after being struck by a camán (the stick used in hurling), which resulted in pneumothorax. The predominant mechanism of injury was running or sprinting (56%, n = 27) and 13% (n = 6) were reported to be overuse injuries. Twelve of the injuries were described as a recurrence of a previous injury (25%, 95% CI 16 to 36%).

**Table 2:** Injury prevalence, context and mechanism in Gaelic games referees

	**Total**		**Football**		**Hurling**		**Dual Football & Hurling**	
	n(%)	95% CI	n(%)	95% CI	n(%)	95% CI	n(%)	95% CI
	**n = 89**		**n = 43**		**n = 22**		**n = 24**	
**Number of referees injured**								
Current gaelic games injury	12 (14)	9 to 21	4 (9)	4 to 19	7 (32)	18 to 49	1 (4)	1 to 17
								
Any injury in past year	54 (61)	52 to 69	30 (70)	57 to 80	11 (50)	33 to 67	13 (54)	38 to 70
Sports injury in past year	50 (56)	47 to 65	27 (63)	50 to 74	11 (50)	33 to 67	12 (50)	34 to 66
Gaelic games-related injury in past year*	46 (52)	43 to 60	25 (58)	46 to 70	11 (50)	33 to 67	10 (42)	27 to 58
								
Context of gaelic games injury (n = 48 injuries) **	**n = 48**		**n = 26**		**n = 11**		**n = 11**	
Refereeing	29 (60)	49 to 71	16 (62)	45 to 76	6 (55)	31 to 76	7 (64)	39 to 83
Training related to refereeing	16 (33)	23 to 45	9 (35)	21 to 51	5 (45)	24 to 69	2 (18)	6 to 43
Linesman or other official Gaelic games duties	3 (6)	3 to 15	1 (4)	1 to 16	--	--	2 (18)	6 to 43
								
**Mechanism of Gaelic games injury (n = 48 injuries)**	**n = 48**		**n = 26**		**n = 11**		**n = 11**	
Contact with player, ball or stick	4 (8)	4 to 17	1 (4)	1 to 16	1 (9)	2 to 32	2 (18)	6 to 43
Running/Sprinting	27 (56)	44 to 67	16 (62)	45 to 76	6 (55)	31 to 76	5 (45)	24 to 69
Turning	7 (15)	8 to 25	3 (12)	5 to 26	1 (9)	2 to 32	3 (27)	11 to 52
Overuse	6 (13)	7 to 22	2 (8)	3 to 21	3 (27)	11 to 52	1 (9)	2 to 32
Unspecified	4 (8)	4 to 17	4 (15)	7 to 30	--	--	--	--
								
**Location of Gaelic games injury. (n = 48 injuries)**	**n = 48**		**n = 26**		**n = 11**		**n = 11**	
Hamstring	12 (25)	16 to 37	9 (35)	21 to 51	2 (18)	6 to 43	1 (9)	2 to 32
Calf	9 (19)	11 to 30	4 (15)	7 to 30	2 (18)	6 to 43	3 (27)	11 to 52
Ankle	4 (8)	4 to 17	3 (12)	5 to 26	--	--	1 (9)	2 to 32
Knee	4 (8)	4 to 17	--	--	2 (18)	6 to 43	2 (18)	6 to 43
Groin	3 (6)	3 to 15	1 (4)	1 to 16	1 (9)	2 to 32	1 (9)	2 to 32
Achilles	4 (8)	4 to 17	2 (8)	3 to 21	2 (18)	6 to 43	--	--
Other lower limb	4 (8)	4 to 17	2 (8)	3 to 21	1 (9)	2 to 32	1 (9)	2 to 32
Back (incl sciatica)	4 (8)	4 to 17	3 (12)	5 to 26	--	--	1 (9)	2 to 32
Head/Neck	2 (4)	1 to 12	1 (4)	1 to 16	--	--	1 (9)	2 to 32
Ribs	2 (4)	1 to 12	1 (4)	1 to 16	1 (9)	2 to 32	-	--

* Includes injury while engaged in refereeing, training related to refereeing, linesman and fitness test

**Two referees had more than one injury, total injuries = 48

All percentages rounded to whole number, so totals may not equal 100%

### Location of Gaelic games related injury

The most common body region injured was the lower limb (n = 40) accounting for 83% of Gaelic games injuries. This translated to an annual prevalence of 45% (95% CI 37 to 54%) for lower extremity injury in the entire cohort. The most prevalent injuries were hamstring strain (n = 12, 25% of injuries) and calf strain (n = 9, 19% of injuries). It was interesting to note that there were 2 injuries to the head and neck and 2 to the ribs, but no upper limb injuries were reported (table 2).

### Impact and severity of injury

Impact and severity of injury was assessed by questions relating to the effect of the injury on ability to referee, train and work as well as limitations experienced in a range of activities of daily living and reported time for the injury to resolve. Twenty eight referees, 31% of the entire group (95% CI 24 to 40%) reported that their Gaelic games injury had been severe enough to prevent them from refereeing. The median time lost from participation in refereeing was 3 weeks, but this ranged widely from 1 to 32 weeks. Thirty nine referees (44% of entire cohort, 95% CI 36 to 53%) indicated that the injury limited their match participation or training regime (table 3). When the injured referees were questioned about whether or not their Gaelic games injury had any impact on their ability to carry out normal activities of daily living, 8 (17%, 95% CI 10 to 28%) reported that their ability to work had been affected, 7 (15%, 95% CI 9 to 26%) stated that ability to drive had been impaired, 20 (44%, 95% CI 32 to 56%) reported that walking was affected, 15 (33%, 95% CI 23 to 45%) said they had problems ascending and descending stairs and 6 (13%, 95% CI 7 to 23%) said they had experienced difficulty in sleeping (table 3).

**Table 3:** Consequences of injury in Gaelic games referees

	**Number of referees**	**% of referees with Gaelic games injury (n = 46)**	**% of all referees (n = 89)**
	n	% (95% CI)	% (95% CI)
Number of referees where injury prevented match participation	28	61 (49 to 72)	31 (24 to 40)
Number of referees experiencing limitation in matches or training	39	85 (74 to 92)	44 (36 to 53)
			
**Number of weeks refereeing or training limited due to Gaelic games injury (n = 39)**			
1	3	7 (3 to 15)	3 (1 to 8)
2–4	23	50 (38 to 62)	26 (19 to 34)
5–8	7	15 (9 to 26)	8 (4 to 14)
9–12	3	7 (3 to 15)	3 (1 to 8)
13–16	2	4 (1 to 12)	2 (1 to 7)
17 +	1	2 (0.5 to 9)	1 (0.3 to 5)
			
Impairment of activity*			
Work	8	17 (10 to 28)	10 (6 to 17)
Driving	7	15 (9 to 26)	8 (4 to 14)
Walking	20	44 (32 to 56)	23 (16 to 31)
Stairs	15	33 (23 to 45)	17 (11 to 24)
Sleeping	6	13 (7 to 23)	7 (4 to 13)

*Multiple effects of injury on activity, percentages do not total 100%

Twelve referees had current ongoing Gaelic games injuries, and for the remaining 34 injured in the past year, there was great variation in the length of time reported for their injuries to resolve ranging from 1 to 24 weeks. The most common recovery period was 3 to 4 weeks with 28% (95% CI 19 to 40%, n = 13) of those with Gaelic games injury in the last 12 months reporting this duration.

### Method of Treatment

Of the 46 referees who sustained a Gaelic games injury in the last 12 months 40 (87%, 95% CI 77 to 93%) consulted a physiotherapist. Four had consulted a doctor for their injury (9%, 95% CI 4 to 18%), and 3 had undergone surgical procedures as a result of injury (7%, 95% CI 3 to 15%). Other treatments methods included medications, injections, oxygen treatment, prescription of orthotics, laser treatment, rest and treatment by a chiropractor. The frequency and financial cost of treatment was not quantified.

## Discussion

The major findings of this study were the amount of refereeing and training that was carried out by elite referees illustrating the professional level of commitment of these individuals to an amateur game, and secondly, the significant Gaelic games related injury burden experienced by this group where the annual prevalence of injury was 58% for football, 52% for hurling and 42% for dual referees.

The year prevalence reported here is somewhat greater than that seen by Bizzini et al. who reported a 40% career (lifetime) prevalence of injury and a 60–63% career prevalence of musculoskeletal complaints in male soccer referees and assistant referees selected for the 2006 FIFA World Cup [5]. The same authors found that career prevalence of injury was 47% and 50% respectively in female FIFA soccer referees and assistant referees [6]. Injury was defined for the soccer referees in accordance with the consensus statement on soccer injury [12] as 'any physical complaint resulting from football match or football training, irrespective of need for medical attention or time loss from football activities'. Thus the higher prevalence found in male Gaelic referees cannot be ascribed to a less stringent injury definition, although injuries occurring during linesman duties and participation in fitness testing were also included in the Gaelic games injury classification used here. The need for a standard definition for injury in Gaelic games is however clear. This should reflect recent published consensus statements in other sports to facilitate comparison of injury statistics between codes.

Given the relationship between ageing, growing lifetime prevalence and susceptibility to chronic or recurrent injury, the latter accounting for 25% injury in these Gaelic games referees, one explanation for the high prevalence noted here may relate to the broad age range of the group (mean age 42 years, range 28 to 55 years). While mean age in male FIFA soccer referees was similar at 41 years, the upper age limit was 10 years younger at 45 years [5] and the female FIFA referees had a mean age of 35 years [6]. The soccer groups studied by Fauno et al. also had lower mean ages (35.3 and 36.5 years) albeit a wide distribution (17 to 65 years) [8].

In Gaelic games, comparison with player data is also limited by differences in injury definition, methodology and duration of data recording, but player reports show a higher incidence rate of injury per individual, 1.72 to 2.2 injuries per player over a season [9,13,14]. The retrospective nature of this study did not allow incidence to be accurately measured, but with 48 injuries in 89 referees, it is clear that the rate (0.54) is far less than one injury per referee over the 12 month period. This compares poorly with the lower injury rate recorded over an entire AFL season by Brukner et al. with 13 injuries in 50 umpires (0.26) [7]. It is not surprising to see fewer injuries per individual in the referee cohort by comparison with players, since they generally do not sustain the same contact forces as the players during match play and do not engage in kicking or high velocity fielding and landing activities. This was further reflected in the absence of hand and upper limb injuries in the referees, injury patterns which are commonly seen in Gaelic footballers and hurlers.

The high frequency of refereeing reflected participation by all of these Gaelic games referees in club as well as elite level duties, a trend that may not be experienced by professional referees in other sports [5,6]. The Gaelic games referees are therefore engaged in greater match play with associated greater injury risk exposure. For Gaelic games referees, mid week club games are common during competition season, while the elite inter-county fixtures are predominantly at weekends, so it is entirely feasible that an individual referee could officiate 5–6 times per week as reported by 9% of the referees surveyed. It emerged that the hurling and dual referees reported particularly high refereeing demands, with 86% of hurling and 88% of dual referees officiating at more than 3 games per week, by comparison with 67% of football referees, possibly reflecting the smaller pool available for hurling. The most common frequency of participation however was 3–4 games per week. Given that a median duration of 3 weeks was reported for one injury to prevent participation in refereeing, it can be extrapolated that should an injury be severe enough to prevent partaking in refereeing duties, a new official has to be found for a minimum of 9 additional games. This time loss from refereeing is in keeping with the findings of Brukner et al. in AFL umpires where an average of 4 weeks absence from games resulted from each injury [7]. Similarly Bizzini et al. found that the most commonly reported absence due to injury was 2–4 weeks in the FIFA male and female soccer referees [5,6]. With 31% (95% CI 24 to 40%) of all Gaelic games referees having such an injury in the past 12 months, the significant impact of referee injury on the organisation of fixtures and allocation of officials can be appreciated. This scenario may, however, under-state the problem as 9% of referees reported that they officiated at 5–6 games on average each week during the season and 18% had refereed more than 12 games in the preceding 4 weeks. Furthermore, the results do not take account of any possible engagement by the referees in linesman, umpire or substitute referee duties which would also be impacted upon following injury.

In common with Gaelic Football players, lower limb injuries were most prevalent, accounting for 83% of Gaelic games injuries in referees (n = 40). This represents a greater proportion of injury than the 69–71% previously reported in Gaelic football players [9,13,14], a trend which may be partly explained by the absence of upper limb injuries in this referee cohort. Similar findings were reported in the previous referee studies where lower limb injury predominated, 85%; (11/13) in AFL umpires, 95% (54/57) in female FIFA referees and 100%, (58/58) in male FIFA referees, while 88% (340/386) soccer referees reported lower extremity soreness in the 1989 study by Fauno et al [8]. The prevalence of lower limb injury translated to 45% (95% CI 37 to 54%) of all Gaelic games referees having a leg injury in the preceding 12 months. It was notable that 10% of the Gaelic refereeing injuries were however sustained to the head, face and ribs, a distribution not seen in the other referee groups [5-7]. Hamstring strains were the most common injury found here (25% of all injuries). This reflects the situation in retrospectively recalled injury for both male (24% injuries) and female (24.5% injuries) FIFA soccer referees [5,6], and also in Gaelic football players as previously reported by Newell et al. [13], where 22% of all incident injuries were to the hamstrings. Calf strains were the second most prevalent injury in the Gaelic games referees (19%), similar to the male (21%) and female (17%) FIFA referees [5,6].

On a personal level, the severity of injury was apparent, with 44% of injured referees having difficulties walking, 33% being limited climbing or descending stairs, 17% reporting interference with ability to work and 13% experiencing impaired sleep due to injury. Eighty seven percent of injured referees consulted a physiotherapist, 9% had a medical consultation and 7% had undergone surgery for their injury. Neither consideration of the costs of treatment nor work loss were within the scope of this survey, but it is clear that there are potential economic consequences of referring injury in Gaelic games and these may warrant further investigation.

One other factor worth noting is the training commitment and exposure of the Gaelic games referees. Frequency of training was recorded here, but precise hours training were not ascertained, limiting direct comparison with FIFA soccer referees where average training durations were in excess of 7 hours per week [5,6]. However, given that 28% (95% CI 21 to 37%) (n = 25) trained 4–5 times and 7% (95% CI 4 to 13%) (n = 6) trained 6–7 times per week during the GAA season, it is likely that some of these Gaelic games referees demonstrated comparable training durations to the elite soccer referees. There was an emphasis on endurance aerobic activities and sprint training in the cohort with only 22% incorporating a strength training component. Combined with the high number of matches this represents a significant exposure to potential injury situations. Sixteen percent of injuries were reported to be due to overuse mechanisms and the relationship between age, exposure, training methods and injury warrants further investigation. It must be acknowledged that data collected here regarding match and training exposure was limited due to the use of average ranges for officiating at games and the focus on frequency of different training components rather than duration of training per week. Caution was therefore exercised in making inferences from the current data and future research should consider a prospective design to record match play and training exposure.

The findings must also be interpreted in the light of possible recall bias, omissions and errors with a retrospective survey methodology. While an 80% response rate may be considered good, injury detail from the other 20% is lacking. Ideally, a prospective study which accurately captures detailed injury data for all referees, with incidence expressed per 1000 hours of exposure to match play and training is required. Validation of injury diagnosis by suitably qualified personnel would strengthen the data accuracy, while the inclusion of a wider referee cohort would provide a more comprehensive picture and allow subgroup comparisons to be made on the basis of level of refereeing, training and age. From this, strategies to guide injury reduction could be developed. These might include approaches such as modification of training schedules, targeted rehabilitation for common recurrent injuries, fitness assessment prior to return to refereeing from injury and use of protective or shock absorbing equipment as appropriate.

## Conclusion

This study is the first to describe the demographic profile and establish the level of refereeing commitment and prevalence of injury in elite Gaelic Games referees. These individuals show immense personal commitment to training for, and participation in Gaelic games, while their injury distributions are seen to mirror those reported for players. Furthermore, the findings suggest that elite Gaelic referees sustain more injuries than their counterparts in soccer and Australian football. Given the pivotal role played by the referee in the games, these findings provide an important insight into the cause, type and consequences of referee injury which impacts on both the referee themselves and on games co-ordination. There are implications for further research into training, prevention and rehabilitation of injury in this group.

## Competing interests

The authors declare that they have no competing interests.

## Authors' contributions

CB was involved in the conception, design, acquisition, analysis and interpretation of data, drafting and final approval of the manuscript. JS was involved in the conception, design, acquisition, analysis and interpretation of data, drafting and final approval of the manuscript. CG was involved analysis and interpretation of data, drafting and final approval of the manuscript.

## Pre-publication history

The pre-publication history for this paper can be accessed here:


